# Preoperative diagnosis of a mediastinal granular cell tumor by EUS-FNA: a case report and review of the literature

**DOI:** 10.1186/1742-6413-2-8

**Published:** 2005-06-08

**Authors:** Sarah M Bean, Mohamad A Eloubeidi, Isam A Eltoum, Robert J Cerfolio, Darshana N Jhala

**Affiliations:** 1 Department of Pathology, Division of Anatomic Pathology, the Department of Medicine, University of Alabama at Birmingham, Birmingham, Alabama, USA; 2 Department of Pathology, Division of Gastroenterology and Hepatology, University of Alabama at Birmingham, Birmingham, Alabama, USA; 3 Department of Cardiothoracic Surgery at the University of Alabama at Birmingham, Birmingham, Alabama, USA

**Keywords:** granular cell tumor, EUS-FNA, cytology, mediastinum

## Abstract

We report the first case of a posterior mediastinal granular cell tumor initially diagnosed on cytologic material obtained via endoscopic ultrasound-guided fine needle aspiration (EUS-FNA) in a 51-year-old male with a prior history of colon cancer. Aspirates obtained were cellular and composed of polygonal cells with abundant granular cytoplasm and small, round dark nuclei. An immunoperoxidase stain performed on the cell block for antibodies to S-100 protein showed strong, diffuse staining of the cytoplasmic granules. Electron microscopy performed on the cell block revealed numerous cytoplasmic lysosomes. This is the first case report in the English literature of a definitive preoperative diagnosis of a mediastinal granular cell tumor utilizing material obtained via EUS-FNA.

## Background

Granular cell tumors are uncommon generally benign soft tissue neoplasms first described in 1926 by Abrikossoff [1]. Granular cell tumors commonly occur in the tongue, gastrointestinal tract, mediastinum, skin, breast, as well as other sites [2-5]. Mediastinal granular cell tumors are, however, exceptionally rare. Fewer than ten cases have been previously described [6-11]. Historically, the diagnosis of mediastinal granular cell tumors has been made on histologic material. Cytologic material was available for only one of the previously described mediastinal granular cell tumors [6]. However, a definitive diagnosis was not rendered based upon the FNA specimen. In this report, we describe the first case report of a preoperative, definitive diagnosis of a mediastinal granular cell tumor diagnosed on a cytologic EUS-FNA specimen. Confirmatory ancillary studies including immunohistochemistry and electron microscopy were performed on the cell block obtained via EUS-FNA.

## Case Presentation

**Figure 1:**
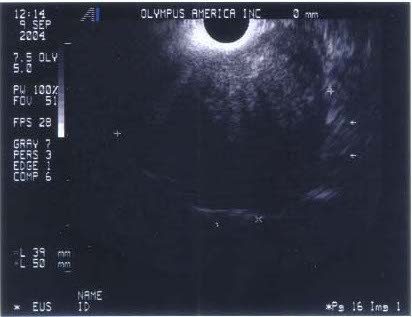
Endoscopic ultrasound image of a 52 x 39 mm hypoechoic mass in the upper mediastinum (Olympus UC-30P).

A 4.9 × 3.1 cm peritracheal posterior mediastinal mass was incidentally discovered on a computed tomography (CT) scan of a 51 year-old gentleman status-post left hemicolectomy in October 2003 for a T3N1Mx, stage III colon cancer. He had moderate gastroesophageal reflux and mild dysphagia. A barium swallow revealed compression and displacement of the proximal thoracic esophagus. Esophageal mucosal ulcerations were not observed. Integrated positron emission tomography(PET)/CT scan using fluorodeoxyglucose (FDG) showed no uptake in the lesion. Due to the benign clinical features of the mass, the differential diagnosis included benign neoplasms such as a leiomyoma or a benign thyroid neoplasm. The possibility of lymphadenopathy secondary to metastatic adenocarcinoma was remote. Endoscopic ultrasound was performed using the UC-30P (Olympus, America, Melville, NY) and revealed a 52- × 39-mm hypoechoic, well-circumscribed posterior mediastinal mass in the thoracic inlet approximately 22 cm from the incisor (Figure 1). After using color Doppler ultrasound, EUS-FNA of the mediastinal mass was performed. Adequate material was obtained for on-site rapid cytopathologic interpretation. The patient underwent a posterior-lateral thoracotomy with entry over the fourth rib. A firm, fixed mass in the posterior mediastinum near the esophagus entering the base of the neck was identified. A 4.0 × 2.8 × 2.5 cm well-circumscribed soft tissue mass was completely resected.

## Materials and methods

For cytologic evaluation, air-dried smears and alcohol-fixed smears were prepared in the endoscopy suite by the cytopathologist and stained with Diff-Quick and Papanicolaou, respectively. Additional material was collected in Hanks balanced salts solution from which a cell block was prepared. Representative histologic sections of the mass and the cell block were fixed in 10% neutral buffered formalin, processed, embedded in paraffin, sectioned to 4 μm thick sections and stained with hematoxylin and eosin.

Immunoperoxidase stains were performed on deparaffinized sections from the cytologic cell block as well as histologic sections in an autostainer. An avidin-biotin-peroxidase complex was used to detect the following antibodies: S-100 (Ventana; predilute), CD68 (Ventana; predilute), thyroglobulin (Cell marque; predilute), and TTF-1 (Dako; 1:50). Electron miscroscopy was performed on the paraffin-embedded cytologic cell block.

The Institutional Review Board at the University of Alabama at Birmingham approved the use of human tissue in this case report.

## Pathologic Findings

**Figure 2:**
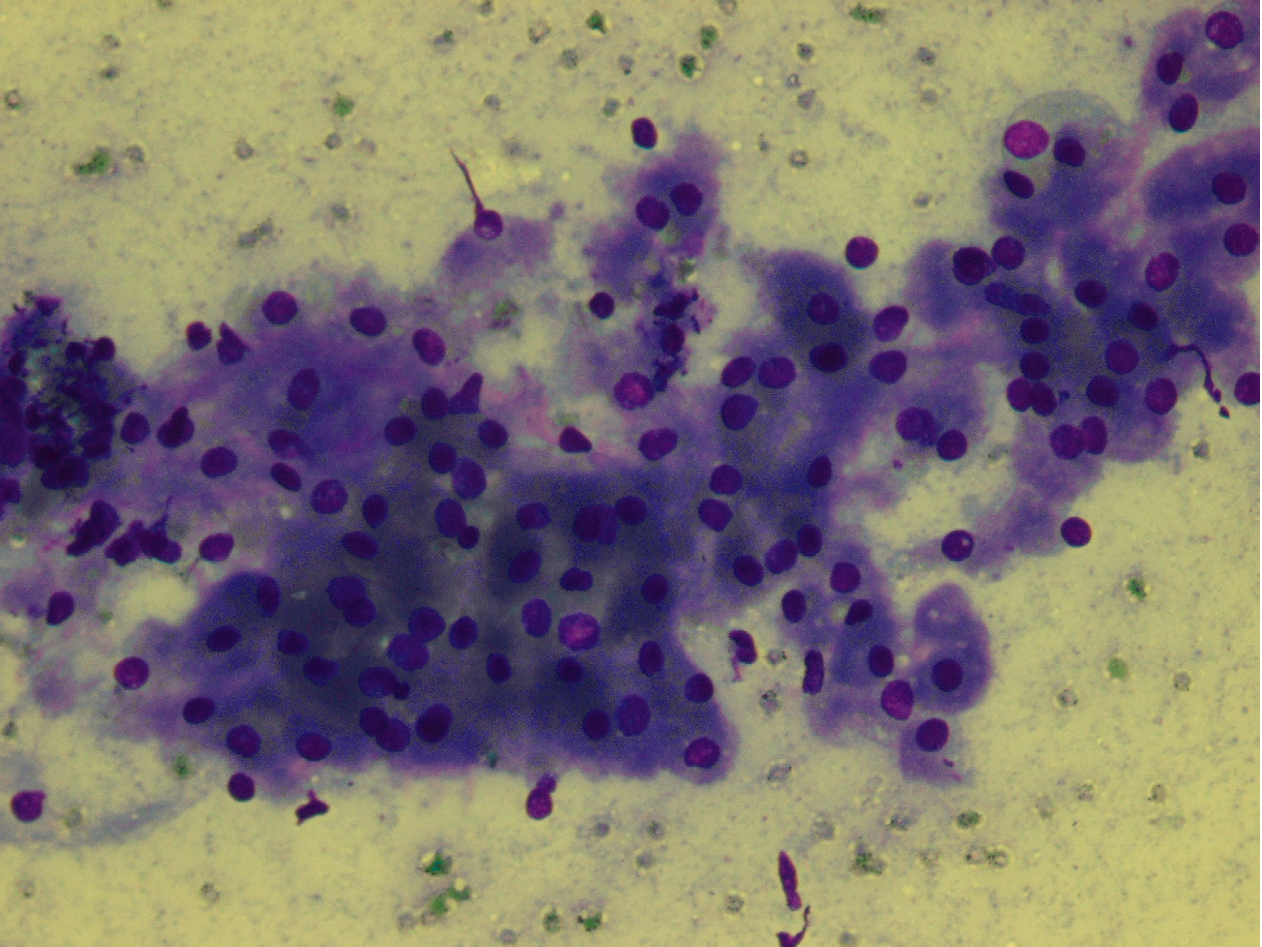
Smears revealed loosely cohesive polygonal cells with small, round, dark nuclei and granular cytoplasm (Diff-Quick, 200X).

**Figure 3:**
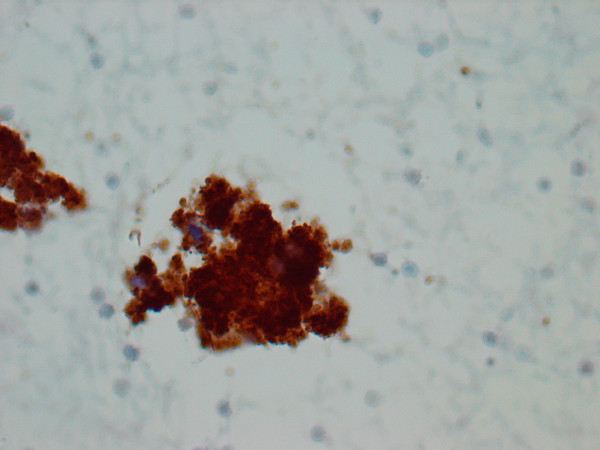
Immunocytochemistry performed on the cell block revealed that the cytoplasmic granules stain strongly for S-100 (400X).

**Figure 4:**
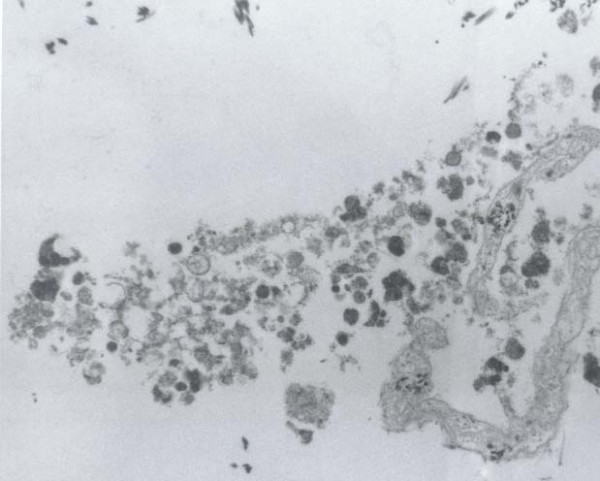
Electron microscopy revealed abundant cytoplasmic lysosomes

The cytologic smears revealed a cellular aspirate composed of loosely cohesive groups of polygonal cells and single cells (Figure 2). The cells had a low N:C ratio with centrally-located small, round, dark nuclei. The cytoplasm contained abundant eosinophilic granules. A background of fine granular debris was also noted. Classification of the lesion was deferred at immediate assessment performed at the time of the procedure. It was noted, however, that diagnostic material had been obtained for ancillary laboratory studies. Immunoperoxidase stains were performed on the cell block with the following results: S-100 (Ventana; predilute) positive, CD68 (Ventana; predilute) positive, thyroglobulin (Cell marque; predilute) negative, and TTF-1 (Dako; 1:50) negative (Figure 3). Positive and negative controls for each stain were appropriately reactive. The morphologic findings and immunohistochemical staining pattern supported a diagnosis of granular cell tumor. Electron microscopy revealed sporadic cells with abundant cytoplasmic pleomorphic electron-dense lysosomes and a relative paucity of organelles characteristic of granular cell tumor (Figure 4).

**Figure 5:**
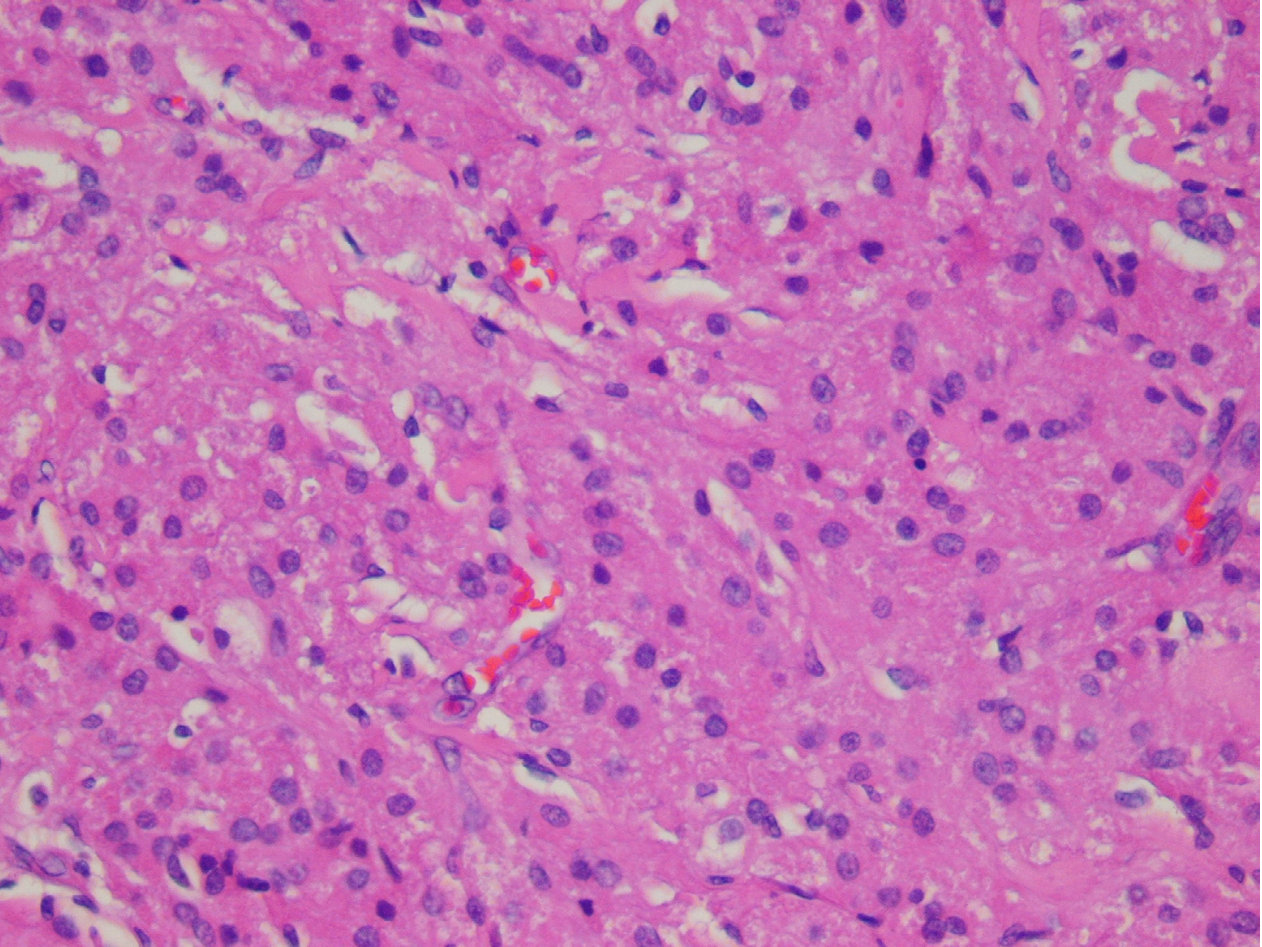
Histologic sections of the resected tumor revealed sheets of polygonal cells with small, round, dark nuclei and granular cytoplasm (200X).

**Figure 6:**
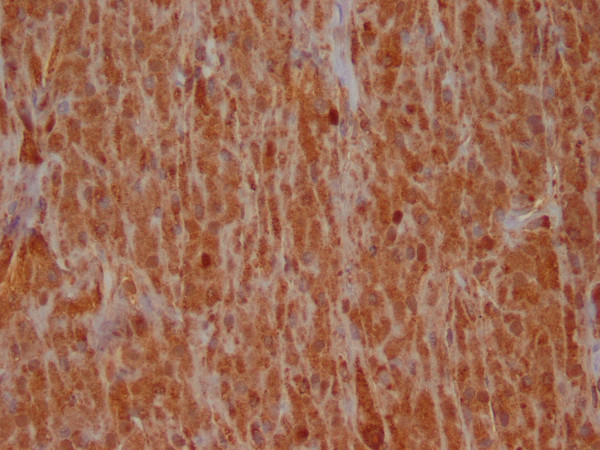
Immunohistochemistry performed on tissue section revealed that the cytoplasmic granules stain diffusely positive for S-100 (200X).

Representative sections of the resected mass were submitted for histologic evaluation. The tumor consisted of sheets of cells similar to those seen in the fine needle aspiration (Figure 5). Frozen section diagnosis of the tumor was granular cell tumor. An immunoperoxidase stain for antibodies to S-100 protein (Ventana; predilute) was performed on a representative section of the formalin-fixed, paraffin embedded tumor. The tumor cells demonstrated diffuse, strong positive cytoplasmic staining with antibodies to S-100 protein (Figure 6), supporting the diagnosis of granular cell tumor.

## Discussion

Mediastinal granular cell tumors are very rare tumors. Prior to this report, only seven mediastinal granular cell tumors have been described in the literature [11]. The average age at diagnosis of those seven patients (including our patient) is 33 years. In all, four men and four women have been diagnosed with mediastinal granular cell tumors. All of the lesions were located in the posterior mediastinum. Four of the patients were asymptomatic, while three patients reported cough, dyspnea, wheezing, and/or chest pain. Our patient experienced mild dysphagia. For this reason, the granular cell tumor was resected. All of these lesions have been surgically resected. In six of the seven reported cases a diagnosis of granular cell tumor was rendered based upon histologic materials.

In 1997, Smith et al. described fine needle aspiration cytology findings of a mediastinal granular cell tumor in a 53 year-old woman [6]. While they described the cytologic findings of a granular cell tumor, they failed to make a definitive, preoperative diagnosis of granular cell tumor utilizing ancillary studies on cytology material. A definitive diagnosis of granular cell tumor was made only after surgical resection and ancillary studies (immunohistochemistry and electron microscopy) were performed on a tissue section.

In the past, mediastinal lesions were sampled utilizing mediastinoscopy, transtracheal biopsy, or CT-guided biopsy. Superior and posterior mediastinal masses have been difficult to approach using those sampling modalities. EUS-FNA, however, has emerged as a safe, effective method of tissue sampling in the mediastinum [12]. Here we describe the first case of a mediastinal granular cell tumor definitively diagnosed based upon EUS-FNA material prior to surgical resection.

The cytologic findings of granular cell tumors have been previously described in organs such as esophagus, breast, tongue, bronchus, and skin [13-15]. Typically smears are composed of large round or polygonal cells with indistinct cytoplasmic borders, abundant PAS positive and S-100 positive cytoplasmic granules, and small round dark eccentric nuclei. In our case, the smears obtained by EUS-FNA were cellular with groups of loosely cohesive sheets and single polygonal to round cells with granular cytoplasm and round, dark nuclei without nucleoli in a background of fine granular debris. Given the anatomic location of the lesion and the morphologic findings, the differential diagnosis included a Hurthle cell neoplasm, benign iatrogenic squamous cells (secondary to trans-esophageal aspiration approach), and granular cell tumor. Metastatic adenocarcinoma, although unlikely, should also be included in the differential diagnosis. The conspicuous lack of nucleoli made a Hurthle cell neoplasm less likely. Furthermore, immunoperoxidase stains for thyroglobulin and TTF-1 were negative, excluding the possibility of a thyroid neoplasm. Iatrogenic squamous cells must be included in the differential diagnosis since the approach of the needle was trans-esophageal. The aspirated cells had distinct polygonal cell borders, a feature of squamous cells. Upon evaluation of the cells at a higher magnification, cytoplasmic granules rather than dense, waxy cytoplasm were apparent, thus, excluding the possibility of squamous cells. Lack of cytoplasmic mucin, columnar cell shape, and nucleocytoplasmic polarity excluded the possibility of a metastatic adenocarcinoma. An S-100 immunoperoxidase stain demonstrated diffuse positive staining of the cytoplasmic granules, consistent with a granular cell tumor. The characteristic morphologic findings in addition to the immunohistochemical staining pattern are diagnostic of a granular cell tumor [16]. The diagnosis was corroborated by findings on electron microscopy and on the surgical resection specimen.

This is the first case report of a preoperative diagnosis of a mediastinal granular cell tumor by EUS-FNA. While granular cell tumors are rare tumors, it is important to include it in the differential diagnosis of mediastinal lesions. The preoperative diagnosis of a granular cell tumor by EUS-FNA will tremendously benefit the patient by preventing further tissue sampling procedures and possibly even surgical resection.

## Competing interests

The author(s) declare that they have no competing interests.

## Authors' contributions

All authors made substantial contributions to the intellectual content and/or presentation of the manuscript. SMB (cytopathology fellow) is the first author, and she wrote the intial version of the manuscript under the guidance of DJ (cytopathologist) who is a senior author. DJ and IE (cytopathologists), diagnosed the cytopathological aspects of the case. ME is the endoscopist who performed the EUS-FNA and revised the manuscript. RC is the surgeon who resected the mass and revised the manuscript.
